# A new treatment method for patients with cancer that cannot be resected en bloc by endoscopic submucosal dissection: the monorail method with clip-line traction

**DOI:** 10.1055/a-2178-4220

**Published:** 2023-10-06

**Authors:** Masashi Ono, Ai Fujimoto, Kazuhisa Yamaguchi, Takahito Toba, Takahisa Matsuda

**Affiliations:** Division of Gastroenterology and Hepatology, Toho University Omori Medical Center, Tokyo, Japan

Endoscopic submucosal dissection (ESD) is a safe endoscopic treatment for early-stage cancer of the esophagus, stomach, duodenum, and colon 1 . However, there are some cases in which ESD is difficult to resect en bloc owing to the location and extent of the lesion, fibrosis, and postoperative effects of the procedure. For these difficult cases, ESD may cause perforation, making en bloc resection difficult, and there are reports of treatment methods using various traction devices 2 3 . The method presented here is to pull the lesion with a clip line, and then pass the thread through a snare from outside the intestinal tract to reach the lesion like a monorail.

The patient was a 73-year-old woman with a 0-IIa lesion measuring almost 100 mm, found in the gastric fornix position. In this case, using the dual knife and Hook knife made the greater curvature side difficult to approach and difficult to detach owing to high respiratory variability. We changed to an IT knife, but most of the lesion was resected, making it difficult to apply tension and difficult to approach, making ESD difficult. Therefore, we first attached a clip line to the lesion ( Fig. 1 ). Traction was applied to the oral side of the lesion. Once the endoscope was removed, the snare was slightly widened from the tip, and a string was inserted through it ( Fig. 2 , Fig. 3 ). The endoscope was inserted through the clip line like a monorail to the lesion ( Fig. 4 ). The snare was opened, and the lesion was resected en bloc while traction was applied with a clip line ( Fig. 5 ). A complete resection with no major complications and residuals was possible ( Video 1 ).

**Fig. 1:**
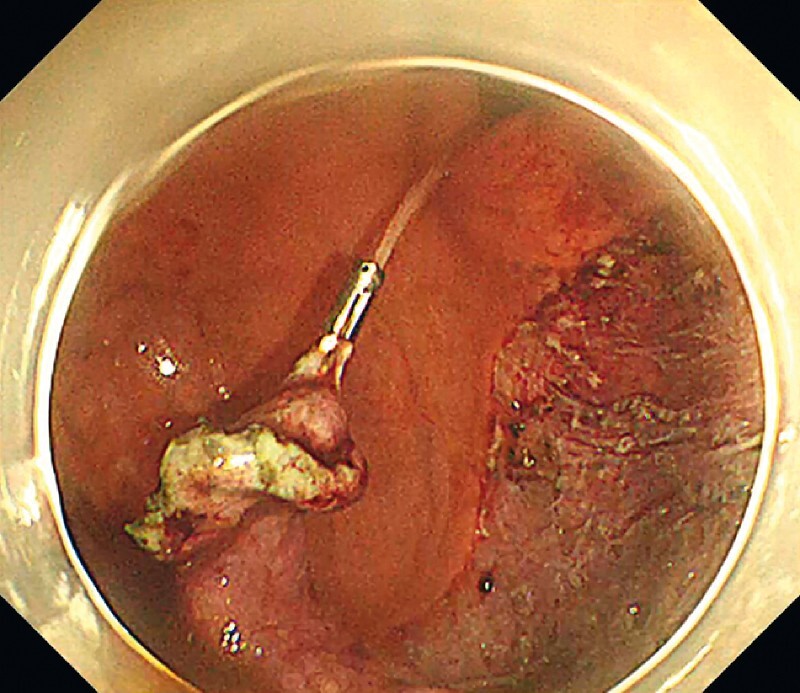
A clip line was attached so traction is applied to the oral side of the lesion.

**Fig. 2:**
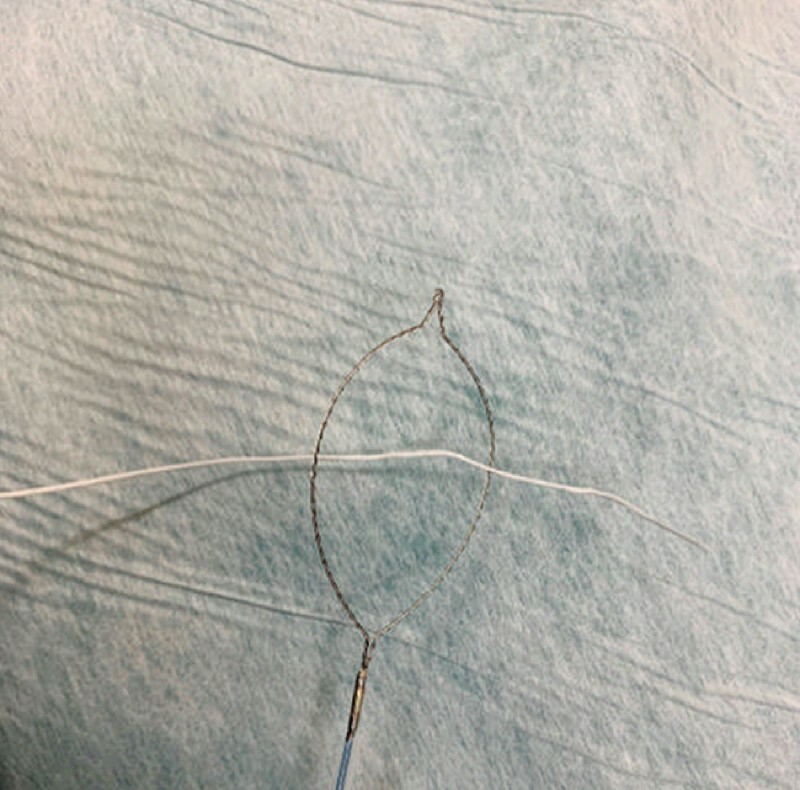
Once the endoscope was removed, the snare was slightly widened from the tip and a string was inserted through it.

**Fig. 3 a:**
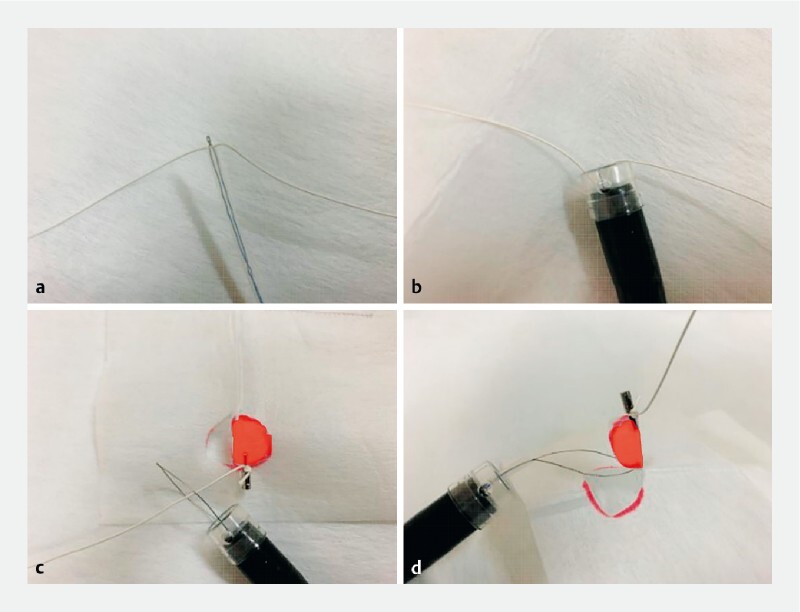
The snare was slightly opened, and a clip line was passed through it. **b** The snare was pulled into the hood at the end of the endoscope. **c** When the lesion was close, the snare was widened. **d** Snaring while pulling the clip line and applying traction.

**Fig. 4:**
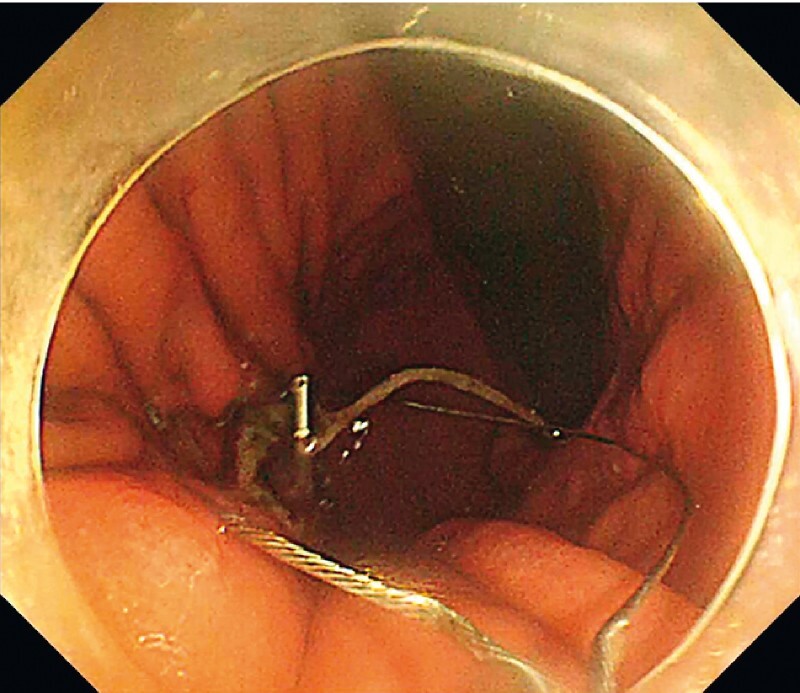
The endoscope was inserted through the clip line like a monorail to the lesion.

**Fig. 5:**
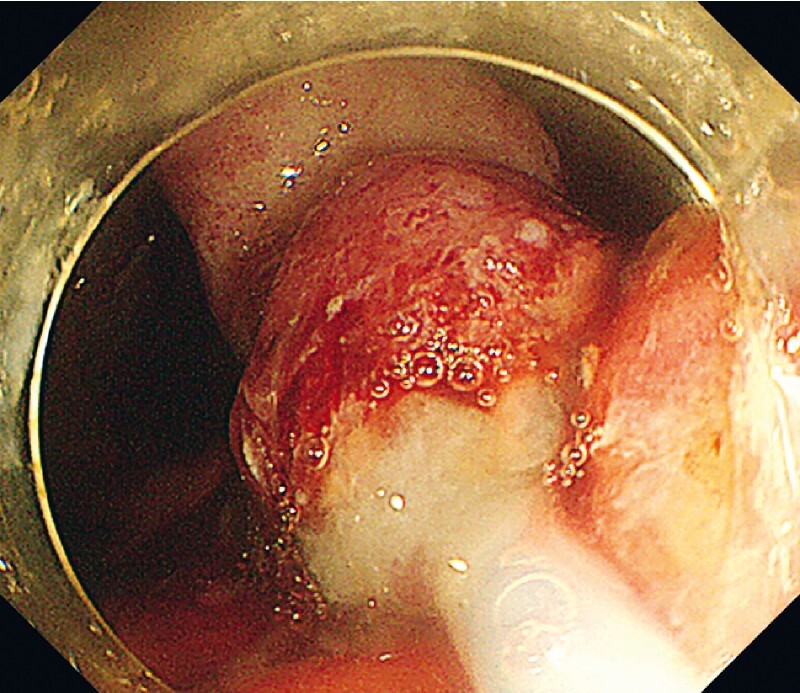
The snare was opened, and the lesion was resected en bloc while traction was applied with a clip line.

**Video 1** A new treatment method for patients with cancer that cannot be resected en bloc by endoscopic submucosal dissection: the monorail method with clip-line traction.


In conclusion, the monorail method allows safe en bloc resection and is considered a new treatment method for difficult ESD cases.

Endoscopy_UCTN_Code_CCL_1AB_2AC_3AB

## Notes

Endoscopy E-Videos

https://eref.thieme.de/e-videos
*E-Videos* is an open access online section of the journal *Endoscopy* , reporting on interesting cases and new techniques in gastroenterological endoscopy. All papers include a high-quality video and are published with a Creative Commons CC-BY license. Endoscopy E-Videos qualify for HINARI discounts and waivers and eligibility is automatically checked during the submission process. We grant 100% waivers to articles whose corresponding authors are based in Group A countries and 50% waivers to those who are based in Group B countries as classified by Research4Life (see: https://www.research4life.org/access/eligibility/ ). This section has its own submission website at https://mc.manuscriptcentral.com/e-videos

## References

[1] Suzuki H Takizawa K Hirasawa T Short-term outcomes of multicenter prospective cohort study of gastric endoscopic resection: ‘Real-world evidence’ in Japan Dig Endosc 2019 31 30 39 30058258 10.1111/den.13246

[2] Abe S Wu S YS Ego M efficacy of current traction techniques for endoscopic submucosal dissection Gut Liver 2020 14 673 684 31887810 10.5009/gnl19266 PMC7667936

[3] Tsuji K Yoshida N Nakanishi H Recent traction methods for endoscopic submucosal dissection World J Gastroenterol 2016 22 5917 5926 27468186 10.3748/wjg.v22.i26.5917 PMC4948268

